# Regional brain volume prior to treatment is linked to outcome after cognitive rehabilitation in traumatic brain injury

**DOI:** 10.1016/j.nicl.2022.103126

**Published:** 2022-07-28

**Authors:** Alexander Olsen, Emily L. Dennis, Jan Stubberud, Elizabeth S. Hovenden, Anne-Kristin Solbakk, Tor Endestad, Per Kristian Hol, Anne-Kristine Schanke, Marianne Løvstad, Sveinung Tornås

**Affiliations:** a Department of Psychology, Norwegian University of Science and Technology, Trondheim, Norway; b Department of Physical Medicine and Rehabilitation, St. Olavs Hospital, Trondheim University Hospital, Trondheim, Norway; c Department of Neurology, University of Utah School of Medicine, Salt Lake City, UT, USA; d George E. Wahlen Veteran Affairs Medical Center, Salt Lake City UT, USA; e Department of Psychology, University of Oslo, Oslo, Norway; f Department of Research, Lovisenberg Diaconal Hospital, Oslo, Norway; g Department of Research, Sunnaas Rehabilitation Hospital, Nesodden, Norway; h RITMO, Department of Psychology, University of Oslo, Norway; i Department of Neurosurgery, Oslo University Hospital – Rikshospitalet, Norway; j Department of Neuropsychology, Helgeland Hospital, 8657 Mosjøen, Norway; k The Intervention Centre, Oslo University Hospital, Oslo, Norway; l Faculty of Medicine, University of Oslo, Oslo, Norway

**Keywords:** Rehabilitation medicine, Magnetic resonance imaging, Brain injury, Executive function, Personalized treatment

## Abstract

Cognitive rehabilitation is useful for many after traumatic brain injury (TBI), but we lack critical knowledge about which patients benefit the most from different approaches. Advanced neuroimaging techniques have provided important insight into brain pathology and systems plasticity after TBI, and have potential to inform new practices in cognitive rehabilitation. In this study, we aimed to identify candidate structural brain measures with relevance for rehabilitation of cognitive control (executive) function after TBI. Twenty-eight patients (9 female, mean age 40.5 (*SD* = 13.04) years) with TBI (>21 months since injury) that participated in a randomized controlled cognitive rehabilitation trial (NCT02692352) were included in the analyses. Regional brain volume was extracted from T1-weighted MRI scans before treatment using tensor-based morphometry. Both positive and negative associations between treatment outcome (everyday cognitive control function) and regional brain volume were observed. The most robust associations between regional brain volume and improvement in function were observed in midline fronto-parietal regions, including the anterior and posterior cingulate cortices. The study provides proof of concept and valuable insight for planning future studies focusing on neuroimaging in cognitive rehabilitation after TBI.

## Highlights

• Advanced neuroimaging has potential to inform new practices in cognitive rehabilitation.
• Regional brain volume was linked to effect of cognitive rehabilitation in traumatic brain injury.
• The most robust effects were observed in midline fronto-parietal brain regions.

## 1 Introduction

Several studies have reported positive effects of cognitive rehabilitation at a group level after acquired brain injury (ABI) (Cicerone et al., 2019, Stamenova and Levine, 2019, Tate et al., 2014, Tornås et al., 2016a), but less is known about which individuals benefit the most from different treatments. Research has indicated that factors such as age and intellectual capacity are non-specific predictors, and that measures of both cognitive and emotional function are mediators of rehabilitation outcome (Tornås et al., 2016b). This points to the relevance of evaluating such factors before assigning patients to cognitive interventions.

Advanced structural and functional neuroimaging methods have provided new insights into brain pathology and system-level plasticity after traumatic brain injury (TBI) (Olsen et al., 2021). Such methods have great potential to generate knowledge about who might benefit from different rehabilitation interventions, but few studies have addressed this in general, and after TBI in particular (Caeyenberghs et al., 2018). To leverage the full potential of imaging methods in cognitive rehabilitation, we need to identify candidate imaging markers that are associated with treatment response. This is a prerequisite for planning large-scale controlled trials (Vander Linden et al., 2018) and in developing tools that may aid clinical decision making and patient stratification (Jenkins et al., 2019).

Despite the heterogeneity in pathology and outcomes after TBI (Maas et al., 2017, Olsen et al., 2021), a growing line of research points to the particular relevance of cognitive control (executive) function, both for real-world functioning and as a target for rehabilitation. Cognitive control dysfunction is common and a significant predictor of poorer everyday functioning, mental health and quality of life after TBI (Azouvi et al., 2017, Finnanger et al., 2015, Spitz et al., 2012). Accordingly, many of the most effective and promising cognitive rehabilitation interventions are based on strengthening the patient's ability to compensate for such difficulties (Stamenova and Levine, 2019, Tate et al., 2014).

Cognitive control functions rely on a dynamic interplay between anatomically wide-spread brain regions (Olsen et al., 2013). Frontal brain regions and white matter tracts, which are important for efficient cognitive control, are particularly susceptible to primary injury in TBI (Bigler, 2001, Bigler and Maxwell, 2011). In addition, secondary injury mechanisms and longer-term processes associated with atrophy and neurodegeneration may lead to further changes in brain structure, even in regions distal to the primary injury (Bigler, 2013, Graham and Sharp, 2019). Cognitive rehabilitation programs focused on cognitive control function are typically administered in the chronic phase after injury (>6 months) when such pathological processes have occurred or are ongoing.

Group-level analyses have shown that certain cortical and subcortical brain regions are more prone to long-term morphometric changes than others (Ledig et al., 2017), indicating common factors despite the heterogeneity in brain pathology and plasticity after TBI. Such common factors may provide a starting point for identifying relevant brain-based markers associated with individual rehabilitation potential. Several studies using magnetic resonance imaging (MRI) have demonstrated a link between morphometric changes in the brain and outcome after TBI (Brezova et al., 2014, Konstantinou et al., 2016), but less is known about the relevance of such measures in informing cognitive rehabilitation. One study found that treatment response to a memory rehabilitation program was associated with volume in fronto-temporal cortices, as well as in the thalamus and the cingulate cortex (Strangman et al., 2010). Interestingly, the authors of this study raised the question whether the observed effects, and particularly those in the cingulate cortex, are specific to memory rehabilitation, or may also extend to rehabilitation of attention and executive functioning (Strangman et al., 2010).

There are a multitude of different approaches to analysis of structural MRI data. In the context of the heterogeneous nature of TBI, tensor-based morphometry (TBM) has some advantages (Dennis et al., 2016, Farbota et al., 2012, Kim et al., 2008, Sidaros et al., 2009). TBM relies on information about the relative position of different brain structures derived from deformation fields. By comparing each individual's brain scan to a common standard template, the deformation fields are used to calculate relative expansion or contraction, and provide measures of regional brain volume. One advantage of TBM is the ability to assess the whole brain, with no need for an *a priori* hypothesis about anatomical regions of interest. TBM does not require accurate gray/white matter segmentation, and can provide measures of brain volume that are more robust than other methods in the context of tissue deformations and contrast changes commonly present after TBI (Kim et al., 2008).

The current study is based on data from an RCT on cognitive rehabilitation of people with ABI, and the results for the primary endpoints have been published elsewhere (Tornås et al., 2016a). Here, we present an analysis of data from a subgroup of patients that participated in this RCT, with a primary goal of identifying candidate structural brain measures with relevance for cognitive control function and rehabilitation after TBI. To this end, we investigated associations between TBM-based regional brain volume and (1) key clinical and cognitive measures before treatment, as well as (2) the subsequent response to cognitive rehabilitation.

## 2 Methods

### 2.1 Participants

This study reports baseline (pre-intervention) and outcome (6 months follow-up) data from a large single-center randomized controlled trial (Tornås et al., 2016a). The study design and participants have been described in detail elsewhere (Tornås et al., 2016a). Briefly, an information letter was sent to 178 potential participants. Persons between 18 and 67 years with a documented non-progressive ABI, at least 6 months post-injury, and ongoing executive impairments, were included. Major psychiatric symptomatology, neurodegenerative disorders, ongoing substance abuse, and/or severe cognitive problems (also including motor function, language comprehension and/or speech impairment) making it difficult to participate in the program were set as exclusion criteria. Ninety persons provided informed consent and underwent a screening interview, 14 declined participation, and 6 did not meet inclusion criteria. Thus, the final sample in the original trial totaled *n* = 70.

Neuropsychological tests and self-reported questionnaires of executive functioning were administered at baseline (pre-intervention), immediately after intervention, and at 6 months follow-up. MRI scans were acquired at baseline. For the specific purpose of the present study, and to obtain control of etiological factors and pathological processes affecting neuroimaging findings, only patients with TBI and available MRI-scans were included. Of the 45 patients with TBI who completed treatment, 34 underwent MRI. All MR data, and the output of each step of the imaging processing were evaluated using visual quality control (QC). Three participants were excluded from the TBM analyses because of lesions/anatomical deformations that were so extensive that the image registration failed (as determined by visual QC), two participants were excluded due to excessive image artifacts, and one was excluded due to missing data, which left a total of 28 participants. All included patients had complicated mild, moderate or severe TBI as determined by Glasgow Coma Scale score (GCS) and radiological findings (MRI/CT). Demographic and injury-related data are presented in Table 1. All participants provided informed consent, and the study was approved by the Regional Committee for Medical Research Ethics (2012/1436, South-Eastern Norway). The study was conducted in accordance with the Helsinki Declaration. Clinical Trial Registration No.: NCT02692352.

**Table 1:** Demographic and brain injury characteristics of the TBI patients.

		Mean	min	max	*SD*
Age		40.5	19	65	13.04
Sex					
	male (%)	19 (67.9 %)			
	female (%)	9 (32.1 %)			
Education, years		13.43	10	18	2.28
Injury mechanism					
Motor vehicle		8 (28.6 %)			
Bicycle		5 (17.9 %)			
Pedestrian		4 (14.3 %)			
Fall		5 (17.9 %)			
Violence		2 (7.1 %)			
Sports injury		2 (7.1 %)			
Other		2 (7.1 %)			
Time since injury, months		121.18	21	575	140.05
Glasgow Coma Scale (GCS)		9.2	3	15	4.68
Acute/subacute clinical CT/MRI findings		28 (100 %)			
Visible lesion on T1w MRI at study baseline		18 (64.3 %)			

Glasgow Coma Scale scores range from 3 (coma) to 15 (fully oriented). GCS from the scene of the accident or at hospital admission in the acute phase was obtained or estimated based on available information in patient records. All included TBI patients had complicated mild, moderate or severe TBI as determined by a Glasgow coma scale score (GCS) and radiological findings. TBI = traumatic brain injury. SD = standard deviation. CT = computed tomography. MRI = magnetic resonance imaging.

### 2.2 Rehabilitation interventions

The participants were randomized to either Goal Management Training (GMT) or the Brain Health Workshop (BHW; Levine et al., 2011). Both interventions were adapted from Levine and colleagues’ manual-based protocols (Levine et al., 2011), translated into Norwegian (Stubberud et al., 2013), and matched regarding hours and intensity of group training, access to educational material, homework, and therapist contact (Tornås et al., 2016a). Briefly, GMT aims to improve executive control in everyday life through the use of attention (e.g., mindfulness) and problem-solving strategies. Participants are taught to stop ongoing behavior using internal cues (“stop-and-think”), to resume supervisory control of cognitive processes and monitor performance. GMT has been tested in various clinical groups with neurological and psychiatric conditions, producing small to medium effect sizes (0.136 - 0.341) on various measures of cognitive control (Boyd et al., 2019, Jensen et al., 2021, Stamenova and Levine, 2019). The BHW involves the use of educational materials and lifestyle topics typically part of psychoeducative ABI rehabilitation programs (Becker et al., 2014). The BHW sessions, and between-session exercises, address topics such as learning about the brain, cognitive (dys)function, stress, physical exercise, sleep, nutrition, and energy management. In the original trial, both the GMT and the BHW group had improvement in self-reported and performance-based cognitive control function (Tornås et al., 2016a, Tornås et al., 2016b).

### 2.3 Performance-based and self-reported function

Performance-based and self-reported function was collected at baseline and follow-up. The Wechsler Abbreviated Scale of Intelligence (WASI; Wechsler, 1999) was applied at baseline to provide an estimate of general intellectual functioning. Cognitive control function is multidimensional and can only partly be captured using performance-based tests (Løvstad et al., 2012). The Behavior Rating Inventory of Executive Function-Adult Version (BRIEF-A; Gioia et al., 2000) was used to measure self-reported control functions in everyday life. It states 75 behaviors to be rated as often, sometimes, or never being a problem over the past 4 weeks. We used the Global Executive Composite (GEC) index, an overarching summary score that incorporates all nine BRIEF-A clinical scales. The BRIEF-A was also used as the primary outcome measure for determining treatment efficacy in the original trial (Tornås et al., 2016a), as well as in the current analyses. A selection of sub-tests from the Delis-Kaplan Executive Function System (D-KEFS; Delis et al., 2001) and Conners’ Continuous Performance Test (CCPT-II; Conners, 2000) were included as performance-based measures of cognitive control (Table 2). Norms from the test manufacturer were used to calculate standardized scores. Two composite scores were computed to provide robust measures of both performance-based cognitive control *efficiency* and *accuracy* (see Table 2). Cognitive control *efficiency* was computed by simple averaging of *T*-scores from response speed-derived measures (Song et al., 2013). Many of the included tests did not provide standardized scores (e.g., *T*-scores) for accuracy. In the original trial (Tornås et al., 2016a), a sum-score of all errors on neuropsychological tests demonstrated some sensitivity to treatment effects. Cognitive control *accuracy* was therefore calculated using the sum of errors from all tests. To provide measures of change, delta scores (Δ) were calculated by subtracting scores at time point 1 (baseline) from scores at time point 2 (post-treatment). Relevant *T*-scores were transformed for consistency in reporting, such that lower scores correspond to poorer performance/more reported problems. Accordingly, positive Δ for measures using *T*-scores correspond to improved function. Δ CC Accuracy reflects the absolute reduction in number of errors, meaning that a negative value corresponds to less errors (improved performance). Mean, *SD*, and Δ are presented in Table 3.

**Table 2:** Performance-based cognitive control efficacy and accuracy composites.

Test	Measure	CC efficacy composite	CC accuracy composite
D-KEFS Trails 4	Time to complete	X	
	Total number of errors		X
D-KEFS CWIT 3	Time to complete	X	
	Total number of errors		X
D-KEFS CWIT 4	Time to complete	X	
	Total number of errors		X
D-KEFS Tower	Time to complete	X	
	Total number of errors		X
CCPT-II	Hit Reaction time	X	
	Omission errors		X
	Commission errors		X
This table shows the test measures included in performance-based cognitive control composite scores. The cognitive control efficacy composite was calculated by averaging *T*-scores (based on norms from the test manufacturer) from the time-based measures. The cognitive control accuracy measure was defined as the sum of all errors across tests. CC = cognitive control. CCPT-II = Conners Continuous Performance Test II. D-KEFS = Delis-Kaplan Executive Function System. CWIT = Color-Word Interference Test.

**Table 3:** IQ, baseline and change (Δ) in cognitive control function with treatment.

Measure	*n*	Mean	*SD*
*Baseline*			
BRIEF GEC	28	34.46	9.00
WASI FSIQ	28	105.07	10.31
CC Efficacy	26	45.42	6.83
CC Accuracy (number of errors)	26	7.77	6.38

*Treatment change (Δ)*			
Δ BRIEF GEC	28	4.71	8.28
Δ CC Efficacy	26	2.28	4.77
Δ CC Accuracy (number of errors)	26	−5.23	11.44

Relevant *T*-scores were transformed for consistency in reporting, such that lower scores = poorer performance/more reported problems. Accordingly, positive Δ for measures using *T*-scores = improved function. Δ CC Accuracy reflects the absolute reduction in number of errors, meaning that a negative value = less errors (improved performance). SD = Standard deviation. CC = Cognitive control. IQ = Intelligence Quotient. BRIEF GEC = Behavior Rating Inventory of Executive Function - Global Executive Composite.

### 2.4 MRI data acquisition

The MRI data were acquired at the Intervention center at Oslo University Hospital using a Phillips Achieva 3 T MRI scanner (Philips, Eindhoven) and an 8-channel head coil. All scans were collected at baseline (>21 months after injury, before treatment). High-resolution structural images were acquired using a T1-weighted multi-shot turbo-field-echo sequence (TR/TE = 6.7/3.1 ms, flip angle = 8°, FOV = 256 × 256 mm, reconstructed into a 256*256 mm matrix with 166 sagittal slices covering the whole brain (voxel size = 1.0 × 1.0 × 1.0) and 0.2 mm slice gap).

### 2.5 Lesion mapping

**Fig. 1:**
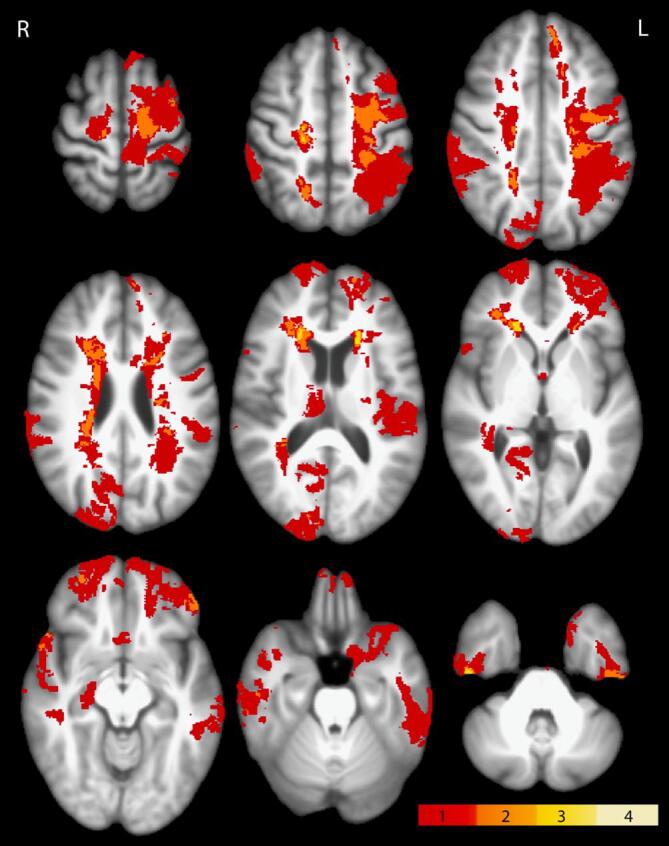
Lesion overlap map. This figure shows the anatomical distribution and overlap of manually segmented lesions visible on the T1 scan. Of the 28 participants included (who all had visible lesions on clinical imaging in the acute/subacute phase), 18 had visible lesions on the baseline (before treatment) T1w scan. Red-yellow scale indicates the degree of overlap between lesions from unique participants.

Initially, the images were evaluated by a radiologist (PKH) to identify positive neuroimaging findings on the baseline scans (Table 1). Before further data processing, visible lesions on T1 images were manually traced and segmented using ITK-SNAP (www.itk snap.org; Yushkevich et al., 2006) by a trained assistant (ESH) and reviewed by an expert in neuroanatomy (ELD). The lesion overlay map is presented in Fig. 1.

### 2.6 Tensor-based morphometry (TBM)

T1-weighted anatomical scans were semi-automatically masked using Brainsuite (https://brainsuite.org/) with manual edits by ELD, and N4-corrected using Advanced Normalization Tools (https://stnava.github.io/ANTs/) to correct for intensity inhomogeneities.

Each participant’s masked, non-uniformity-corrected, template-aligned T1-weighted image was aligned to the MNI template, using ANTs for rigid, affine, and non-linear registration. Symmetric Normalization (SyN; Avants et al. 2008) registration used a multi-level approach, i.e., the ‘moving’ and fixed T1-weighted images were successively less smoothed at each level, with a full resolution registration occurring at the final level. We used 1000, 500, 250 and 100 iterations at each level, with a Gaussian kernel smoothing sigma set to 3, 2, 1 and 0, respectively (7.05, 4.7, 2.35 and 0 voxels full width at half maximum) and shrink factors of 8, 4, 2 and 1, respectively. Image similarity was measured using the ANTs implementation of mutual information (Avants et al. 2011). The lesion maps, registered to MNI space using the warp fields from the T1 registration above, were included in the registration using the -x flag. Image intensities were winsorized, excluding top and bottom one percent of voxels, and histogram matching was used. The output Jacobian determinant image showed the direction and magnitude of volume difference between the participant’s T1 and the template.

### 2.7 Statistical analyses

In our voxel-wise linear regression testing for associations with clinical and cognitive variables, we did not include intracranial volume (ICV) as a covariate. The rigid and affine registrations that were part of our processing protocol account for differences in overall brain scale, removing much of the effect of ICV. Moreover, many prior analyses have not found statistical differences when ICV was included as a covariate (King et al., 2020, Miller et al., 2022). To examine associations between regional brain volume and the primary outcome measure from the rehabilitation trial (BRIEF-As GEC score; BRIEF-GEC), we tested the following model:

$$X=A+\beta 1BRIEF_{\mathrm{change}}+\beta 2Age+\beta 3Sex+\varepsilon$$

where *X* is the Jacobian determinant value at a given position, *A* is the constant Jacobian determinant term, the βs are the regression coefficients for the variable of interest and covariates, and ε is an error term. Additionally, to account for some of the heterogeneity in our sample and aid interpretation of findings, we tested a more conservative model which was adjusted for baseline cognitive control functioning (BRIEF-GEC), injury severity (GCS), and time since injury (TSI). Secondary analyses also tested for associations between baseline measures (GCS, TSI, IQ, BRIEF-GEC, CC efficacy, CC accuracy), as well as Δ CC efficacy and Δ CC accuracy. We used the ‘lm()’ function from the ‘stats’ package in R (https://stat.ethz.ch/R-manual/R-devel/library/stats/html/lm.html, version 2.9.2) to fit each model using linear regression voxel-wise. For each model, results were corrected for multiple comparisons across all voxels tested using Searchlight FDR [false discovery rate], *q* < 0.05 (Langers et al., 2007). Searchlight FDR uses a sliding window approach to correct for multiple comparisons, yielding improved sensitivity over conventional FDR while maintaining the specificity of conventional FDR and FWE (family-wise error) approaches. We report clusters exceeding 50 voxels only. Covariates across the models included age and sex.

## 3 Results

**Fig. 2:**
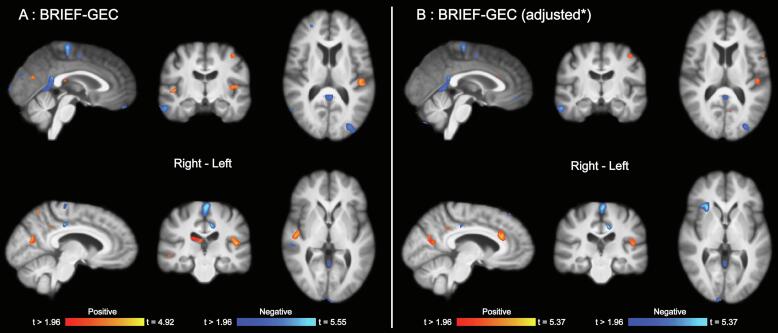
Associations between baseline (before treatment) regional brain volume and BRIEF-GEC score change (Δ). Associations between baseline regional brain volume and BRIEF-GEC score change (Δ), *adjusted for baseline BRIEF-GEC, GCS and TSI. Age and sex were also included as covariates in the model. Analyses were corrected for multiple comparisons across all voxels tested using Searchlight FDR [false discovery rate], *q* < 0.05 (Langers et al., 2007). BRIEF-GEC = Behavior Rating Inventory of Executive Function - Global Executive Composite. GCS = Glasgow coma scale. TSI = Time since injury.

**Fig. 3:**
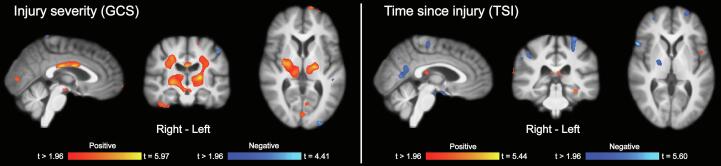
Associations between baseline (before treatment) regional brain volume, GCS and TSI. Associations between baseline regional brain volume, injury severity (GCS) and time since injury (TSI). Age and sex were included as covariates in the model. Analyses were corrected for multiple comparisons across all voxels tested using Searchlight FDR [false discovery rate], *q* < 0.05 (Langers et al., 2007). GCS = Glasgow coma scale. TSI = Time since injury.

**Fig. 4:**
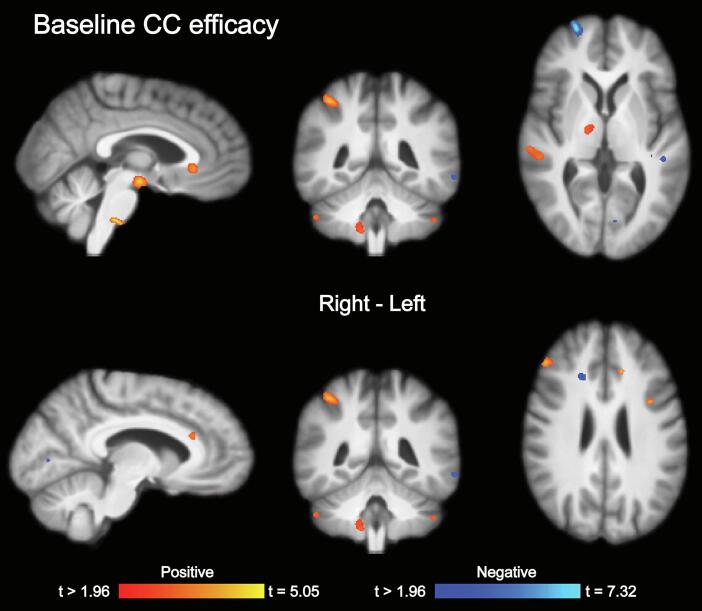
Associations between baseline (before treatment) regional brain volume and baseline cognitive control efficacy. Associations between baseline regional brain volume and baseline cognitive control (CC) efficacy. Age and sex were included as covariates in the model. Analyses were corrected for multiple comparisons across all voxels tested using Searchlight FDR [false discovery rate], *q* < 0.05 (Langers et al., 2007).

We found that regional brain volume at baseline was significantly associated with treatment outcome (Fig. 2**,** Table 4, Table 5). In the main (unadjusted) analysis, larger regional brain volumes in widespread areas including parietal-, occipital-, and temporal cortices, subcortical regions, and the cerebellum, were associated with larger gains on the BRIEF-GEC score, i.e. self-reported everyday cognitive control. The more conservative model adjusting for baseline BRIEF-GEC score, injury severity (GCS), and time since injury (TSI) generally revealed very similar results, but with less significant effects in regions adjacent to the ventricles (i.e., adjacent to the thalamus), and more pronounced effects in cortical regions, especially those encompassing anterior and posterior cingulate cortices, as well as midline parietal regions. Of note, the unadjusted analysis also showed significant associations between lower regional brain volume and positive gain on the BRIEF-GEC in widespread regions. Notably, some of the larger clusters were adjacent to- or overlapping with regions with cerebrospinal fluid (CSF) (ventricles, major sulci). The adjusted model (baseline BRIEF-GEC, GCS, TSI) generally revealed similar, but less pronounced, negative associations, except of showing an additional significant cluster in the right insula. There were significant effects of both injury severity (GCS) and time since injury (TSI), but these demonstrated generally low degrees of anatomical overlap with the clusters associated with treatment outcome (Fig. 3**,** Table 6, Table 7). Higher GCS score (less severe injury) was primarily associated with significant clusters of larger regional brain volume in key white matter tracts (e.g., corpus callosum, corticospinal tract), cortical regions, thalamus, and brainstem. There were only a few very small clusters of significant associations between higher GCS (less severe injury) and lower brain volume. Time since injury was primarily associated with lower regional brain volume in frontoparietal cortical regions, areas in the temporal lobe, as well as subcortical structures (e.g., globus pallidus). Higher CC efficacy at baseline was primarily associated with larger regional brain volume in cortical and subcortical brain areas, but also a few small clusters of lower regional brain volume (Fig. 4**,** Table 8). None of the other models yielded statistically significant results.

**Table 4:** Associations between baseline (before treatment) regional brain volume and BRIEF-GEC score change (Δ).

			MNI coordinates (peak)				
Anatomical region (cluster peak)	Size (number of voxels)	t (max)	X	Y	Z	R/L	Tissue
*Positive associations*							
Lateral ventricles	1606	3,81	−2	−28	16	L	CSF
Cerebellum	1362	4,17	47	−64	−32	R	GM
Precuneus	1186	4,2	−13	−43	35	L	WM
Superior temporal gyrus	967	4,36	50	−12	0	R	WM
Cerebellum	907	4,04	12	−87	−44	R	GM
Transverse temporal gyrus	872	4,25	−44	−21	13	L	GM
Cuneus	736	3,89	−6	−73	17	L	GM
Cerebellum	679	4,92	−38	−64	−28	L	GM
Insula	359	3,64	−38	−10	5	L	GM
Lingual gyrus	355	4,89	−20	−74	−7	L	GM
Superior parietal lobule	313	3,81	−31	−35	44	L	WM
Superior parietal lobule	274	4,33	16	−64	46	R	WM
Angular gyrus	250	3,79	−27	−50	35	L	GM
Precentral gyrus	235	3,64	−39	−11	52	L	GM
Supramarginal gyrus	139	3,68	−62	−33	31	L	GM
Supramarginal gyrus	123	4,22	39	−41	32	R	WM
Precuneus	111	3,75	−9	−63	56	L	GM
Posterior thalamic radiation	98	3,48	35	−53	7	R	WM
Postcentral gyrus	90	3,89	56	−15	34	R	GM
Lateral occipital gyrus	77	4,1	−39	−69	30	L	GM

*Negative associations*							
Fusiform gyrus	5540	4,05	36	−31	−28	R	GM
Precentral gyrus	1612	5,8	−2	−22	66	L	GM
Posterior cingulate gyrus	1529	4,58	3	−43	20	R	GM
Lateral occipital gyrus	1346	4,43	−30	−91	15	L	GM
Middle temporal gyrus	929	4,29	64	−5	−26	R	GM
Cuneus	540	4,35	−1	−89	25	L	GM
Postcentral gyrus	394	4,45	34	−32	65	R	GM
Cingulate gyrus	376	5,55	−9	−23	38	L	GM
Superior temporal gyrus	308	3,96	54	−28	−1	R	GM
Superior parietal lobule	221	4	−22	−68	55	L	GM
Middle frontal gyrus	175	3,5	39	34	32	R	GM
Supramarginal gyrus	129	3,62	54	−31	33	R	GM
Precentral gyrus	76	4,2	55	6	39	R	GM
Insula	54	3,41	28	28	4	R	GM
Superior frontal gyrus	52	3,83	27	60	11	R	GM

Associations between baseline regional brain volume and BRIEF-GEC score change (Δ). Analyses were corrected for multiple comparisons across all voxels tested using Searchlight FDR [false discovery rate], *q* < 0.05 (Langers et al., 2007). Only clusters exceeding 50 voxels are reported. Age and sex were included as covariates in the model. Note that some clusters are relatively large and therefore span over several brain regions (see Fig. 2 for details). BRIEF-GEC = Behavior Rating Inventory of Executive Function - Global Executive Composite. MNI = Montreal Neurological Institute. R/L = Right/Left. GM = Gray matter. WM = White matter. CSF = Cerebrospinal fluid.

**Table 5:** Associations between baseline (before treatment) regional brain volume and BRIEF-GEC score change (Δ), adjusted for baseline BRIEF-GEC, GCS and TSI.

			MNI coordinates (peak)				
Anatomical region (cluster peak)	Size (number of voxels)	t (max)	X	Y	Z	R/L	Tissue
*Positive associations*							
Cerebellum	1612	4,26	34	−69	−28	R	GM
Cingulate gyrus/Cingulum	1327	4,66	−7	35	24	L	GM/WM
Lateral ventricles	968	4,1	25	−38	19	R	CSF
Superior parietal lobule	929	5,42	−29	−36	46	L	WM
Cerebellum	707	4,02	12	−87	−43	R	GM
Cerebellum	688	4,99	−38	−63	−29	L	GM
Precuneus	588	3,57	−10	−43	36	L	WM
Cuneus	540	3,97	−6	−71	23	L	GM
Transverse temporal gyrus	455	3,99	−45	−20	13	L	GM
Lingual gyrus	358	4,56	−20	−74	−8	L	GM
Precentral gyrus	305	3,55	−50	−14	51	L	GM
Supramarginal gyrus	195	4,51	40	−41	32	R	WM
Posterior thalamic radiation	115	3,49	35	−54	7	R	WM
Lateral occipital gyrus	88	3,9	−39	−69	30	L	GM
Superior parietal lobule	83	4,6	−20	−69	45	L	GM
Inferior frontal gyrus	52	3,94	−54	16	12	L	GM

*Negative associations*							
Fusiform gyrus	4044	4,61	38	−53	−12	R	GM
Insula	1177	5,37	28	30	1	R	GM
Posterior cingulate gyrus	1123	4,17	3	−43	20	R	GM
Middle temporal gyrus	924	4,33	63	−6	−26	R	GM
Insula	853	4,36	38	1	−11	R	GM
Precentral gyrus	832	5,09	0	−21	64	R	GM
Lateral occipital gyrus	820	3,82	−30	−91	15	L	GM
Superior frontal gyrus	528	3,74	−1	−2	53	L	GM
Superior frontal gyrus	462	4,52	−15	45	52	L	GM
Superior frontal gyrus	284	4,51	−15	22	48	L	WM
Middle frontal gyrus	281	4,02	−26	18	57	L	GM
Cingulate gyrus	228	4,99	−10	−24	36	L	GM
Inferior rostral gyrus	218	3,62	−2	60	−9	L	GM
Inferior fronto-occipital fasciculus	187	4,39	24	4	−9	R	WM
Middle frontal gyrus	137	3,31	38	34	32	R	GM
Superior parietal lobule	123	3,63	−24	−68	54	L	GM
Cuneus	109	3,8	0	−89	25	R	GM
Precentral gyrus	76	4,11	56	7	40	R	GM

Associations between baseline regional brain volume and BRIEF-GEC score change (Δ), adjusted for baseline BRIEF-GEC, GCS and TSI. Age and sex were also included as covariates in the model. Analyses were corrected for multiple comparisons across all voxels tested using Searchlight FDR [false discovery rate], *q* < 0.05 (Langers et al., 2007). Only clusters exceeding 50 voxels are reported. Note that some clusters are relatively large and therefore span over several brain regions (see Fig. 2 for details). BRIEF-GEC = Behavior Rating Inventory of Executive Function - Global Executive Composite. GCS = Glasgow coma scale. TSI = Time since injury. MNI = Montreal Neurological Institute. R/L = Right/Left. GM = Gray matter. WM = White matter. CSF = Cerebrospinal fluid.

**Table 6:** Associations between baseline (before treatment) regional brain volume and injury severity (GCS).

			MNI coordinates (peak)				
Anatomical region (cluster peak)	Size (number of voxels)	t (max)	X	Y	Z	R/L	Tissue
*Positive associations*							
CC/CR/IC	20,371	5,97	21	−8	−1	R	WM
CC/CR/IC	8004	5,46	−19	−13	7	L	WM
Precentral gyrus	1358	4,15	−10	−21	63	L	GM
Inferior temporal gyrus	679	3,73	40	−14	−37	R	GM
Superior parietal lobule	626	3,72	30	−40	67	R	GM
Lateral occipital gyrus	441	4,49	27	−73	16	R	WM
Lingual gyrus	418	4,68	−22	−65	−9	L	GM
Superior parietal lobule	399	3,31	−27	−54	63	L	GM
Cuneus	361	3,73	9	−65	11	R	GM
Cuneus	295	3,7	1	−88	5	R	GM
Lingual gyrus	273	3,86	−6	−72	2	L	GM
Precentral gyrus	267	3,63	−56	2	21	L	GM
Superior parietal lobule	236	3,48	−33	−42	38	L	GM
Lateral occipital gyrus	204	4,19	−30	−71	32	L	GM
Lingual gyrus	188	3,56	23	−51	−14	R	GM
Fusiform gyrus	184	3,16	−50	−63	−22	L	GM
Cerebellum	156	3,73	10	−37	−18	R	GM
Angular gyrus	63	3,59	33	−45	31	R	WM

*Negative associations*							
Middle occipital gyrus	588	4,06	−24	−99	3	L	GM
Postcentral gyrus	412	4,02	−49	−15	48	L	GM
Temporal pole	376	4,05	31	20	−41	R	GM
Posterior orbital gyrus	272	3,82	−26	32	−11	L	WM
Middle temporal gyrus	219	3,58	−53	−64	23	L	GM
Inferior occipital gyrus	198	4,18	−36	−79	−4	L	GM
Angular gyrus	162	4,41	32	−67	34	R	GM
Posterior thalamic radiation	138	3,5	49	−47	−2	R	WM
Superior temporal gyrus	120	3,51	−45	−33	3	L	GM
Middle frontal gyrus	87	3,39	−23	39	41	L	GM

Associations between baseline regional brain volume and injury severity (GCS). Age and sex were included as covariates in the model. Analyses were corrected for multiple comparisons across all voxels tested using Searchlight FDR [false discovery rate], *q* < 0.05 (Langers et al., 2007). Only clusters exceeding 50 voxels are reported. Note that some clusters are relatively large and therefore span over several brain regions (see Fig. 3 for details). GCS = Glasgow coma scale. MNI = Montreal Neurological Institute. R/L = Right/Left. GM = Gray matter. WM = White matter.

**Table 7:** Associations between baseline (before treatment) regional brain volume and time since injury (TSI).

			MNI coordinates (peak)				
Anatomical region (cluster peak)	Size (number of voxels)	t (max)	X	Y	Z	R/L	Tissue
*Positive associations*							
Superior temporal gyrus	1214	4,86	48	−17	−6	R	GM
Medial orbitofrontal cortex	1144	4,4	19	60	−13	R	GM
Lateral ventricles	1005	4,28	−7	−25	12	L	CSF
Precuneus	567	5,44	−14	−54	68	L	GM
Middle temporal gyrus	540	4,71	−57	−45	−7	L	GM
Angular gyrus	515	3,95	−32	−62	44	L	GM
Parietal operculum	350	4,73	−35	−21	17	L	GM
Fusiform gyrus	265	3,98	−31	−32	−15	L	GM
Superior frontal gyrus	227	3,76	−7	68	35	L	GM
Middle temporal gyrus	202	3,31	−53	3	−36	L	GM
Superior frontal gyrus	194	4,92	−8	57	19	L	GM
Insula	90	4,41	−38	−9	−6	L	GM
Inferior frontal gyrus	74	3,47	−34	9	11	L	GM
Superior frontal gyrus	69	3,41	−18	15	45	L	WM
Middle frontal gyrus	68	3,77	28	29	49	R	GM

*Negative associations*							
Precentral gyrus	3257	5,35	−19	−19	60	L	GM
Lingual gyrus	2161	5,6	9	−64	10	R	GM
Precentral gyrus	1903	4,74	24	−25	51	R	WM
Entorhinal cortex	1357	4,24	15	3	−23	R	GM
Superior frontal gyrus	770	5,38	−25	75	9	L	GM
Angular gyrus	736	5,45	−35	−44	37	L	GM
Superior frontal gyrus	704	3,91	−8	73	26	L	GM
Globus pallidus	642	4,11	22	−3	0	R	GM
Angular gyrus	580	4,01	56	−52	29	R	GM
Inferior frontal gyrus	504	5,19	55	25	4	R	GM
Cuneus	484	3,9	−1	−95	17	L	GM
Medial orbitofrontal cortex	468	4,28	−13	50	−9	L	WM
Angular gyrus	429	4,07	43	−29	41	R	GM
Lingual gyrus	313	3,78	−20	−64	−4	L	GM
Cerebellum	285	3,85	34	−51	−32	R	GM
Supramarginal gyrus	215	3,75	55	−32	36	R	GM
Superior frontal gyrus	92	3,31	16	−4	59	R	WM
Middle frontal gyrus	82	3,65	29	63	4	R	GM

Associations between baseline regional brain volume and time since injury (TSI). Age and sex were included as covariates in the model. Analyses were corrected for multiple comparisons across all voxels tested using Searchlight FDR [false discovery rate], *q* < 0.05 (Langers et al., 2007). Only clusters exceeding 50 voxels are reported. Note that some clusters are relatively large and therefore span over several brain regions (see Fig. 3 for details). GCS = Glasgow coma scale. MNI = Montreal Neurological Institute. R/L = Right/Left. GM = Gray matter. WM = White matter.

**Table 8:** Associations between baseline (before treatment) regional brain volume and baseline cognitive control (CC) efficacy.

			MNI coordinates (peak)				
Anatomical region (cluster peak)	Size (number of voxels)	t (max)	X	Y	Z	R/L	Tissue
*Positive associations*							
Superior temporal gyrus	1879	5,05	53	−32	0	R	GM
Superior parietal lobule	1155	4,58	38	−43	51	R	GM
Cerebellum	633	3,72	13	−51	−48	R	GM
Cingulate gyrus	575	3,88	6	34	−2	R	GM
Middle frontal gyrus	429	4,46	45	45	31	R	GM
Middle frontal gyrus	357	4,24	41	25	49	R	GM
Thalamus	337	3,18	16	−8	4	R	GM
Middle cerebellar peduncle	287	4,82	4	−26	−44	R	WM
Cingulate gyrus	277	4,32	−12	36	26	L	GM
Middle frontal gyrus	187	4,37	−35	13	29	L	GM
Cerebellum	128	3,33	49	−49	−41	R	GM
Caudate	127	3,64	−13	25	8	L	GM
Inferior occipital gyrus	110	3,54	−28	−97	−14	L	GM

*Negative associations*							
Superior temporal gyrus	890	5,27	46	−40	15	R	GM
Superior frontal gyrus	655	7,32	24	73	3	R	GM
Superior frontal gyrus	501	5,4	10	42	55	R	GM
Lingual gyrus	453	4,09	−14	−82	−9	L	GM
Middle frontal gyrus	241	5,06	28	26	49	R	GM
Superior temporal gyrus	194	4,43	−45	−31	1	L	GM
Superior corona radiata	150	3,26	19	33	29	R	WM
Middle temporal gyrus	57	3,66	−62	−46	−10	L	GM

Associations between baseline regional brain volume and baseline cognitive control (CC) efficacy. Age and sex were included as covariates in the model. Analyses were corrected for multiple comparisons across all voxels tested using Searchlight FDR [false discovery rate], *q* < 0.05 (Langers et al., 2007). Only clusters exceeding 50 voxels are reported. Note that some clusters are relatively large and therefore span over several brain regions (see Fig. 3 for details). GCS = Glasgow coma scale. MNI = Montreal Neurological Institute. R/L = Right/Left. GM = Gray matter. WM = White matter.

## 4 Discussion

This study shows that measures of brain structure obtained before treatment are associated with cognitive rehabilitation outcomes. Both positive and negative associations between outcome and regional brain volume in a wide range of anatomical locations were observed. The most pronounced associations between larger TBM-based regional brain volume and positive outcome were found in midline fronto-parietal cortical regions, including the anterior and posterior cingulate cortices which are known to be key areas for cognitive control processing in the general population (Olsen et al., 2013), and functionally altered after TBI (Olsen et al., 2015). These effects did not overlap with visible lesions or general injury related effects (i.e., GCS, TSI). The most pronounced associations between lower TBM-based regional volume and positive outcome were primarily observed in areas adjacent to- or overlapping with non-brain regions, including CSF (e.g., along ventricles and major sulci), which are known to be susceptible to morphometric changes caused by atrophy or neurodegeneration after TBI (Graham and Sharp, 2019, Kim et al., 2008).

When adjusting for baseline self-reported cognitive control function, injury severity (GCS score), and time since injury, the effects observed in midline cortical regions generally increased in strength. However, effects observed in areas adjacent to or overlapping with regions with CSF were reduced, which further indicates a dissociation in the underlying mechanisms causing the respective findings. One interpretation may be that effects found in midline cortical regions reflect preserved capacity for cognitive control processing which facilitates positive treatment response, whereas the effects observed in regions adjacent to or overlapping with CSF potentially reflect more general injury related factors. Further pointing to their functional relevance for rehabilitation, the effects observed in midline cortical regions partly overlapped with regions that have been associated with outcome after a memory rehabilitation program in a group of TBI patients of all severities (Strangman et al., 2010). Brain volume in anatomical regions typically considered to be more specifically linked to memory function, such as the hippocampus, also predicted outcome after memory rehabilitation (Strangman et al., 2010). However, interestingly, the effects in the midline cortical region not only predicted specific outcomes (verbal list learning task), but also more general ecologically valid outcomes (everyday memory functioning). In the context of our own findings, this may indicate that these brain areas play a more domain general role which benefits a wider range of cognitive rehabilitation settings.

There is no obvious explanation for associations between reduced brain volume because of injury and improved outcome, as the opposite would typically be expected. This may be a random observation, but possibly also reflect methodological limitations of TBM. TBM aggregates information of expansion or contraction over a small region that may cross micro-scale tissue boundaries, and it is important to note that TBM provides information on the regional volume deformations and not the integrity of the brain tissue per se. Brain segmentation in these areas is also challenging, especially in the context of TBI (Ledig et al., 2017), and an alternative explanation of the results may be that the TBM-based measure is partly reflecting lower CSF volume, e.g., due to less atrophy or neurodegeneration. This interpretation is also supported by the observation that some of these clusters were adjacent to regions that showed lower regional brain volume with increasing time since injury. Future studies using advanced multimodal MRI techniques are needed to provide even more precise separation of effects across tissue types (Natu et al., 2019).

There was considerable heterogeneity regarding injury severity in this study, and GCS scores ranged from 3 to 15. In an analysis investigating the effects of injury severity directly, we observed limited anatomical overlap with the effects related to positive rehabilitation outcomes. However, more severe injury (lower GCS score) was linked to large clusters of lower regional brain volume in cortical regions, key white matter tracts (e.g., corpus callosum, corticospinal tract), as well as in the thalamus and brainstem. More severe TBI is linked to a larger degree of traumatic axonal injury (TAI) in the corpus callosum and the brain stem, which in turn is linked to poorer outcomes (Skandsen et al., 2020). Both primary and secondary injury mechanisms affecting the thalamus are also typically found in more severe TBI and are associated with poorer outcomes (Lutkenhoff et al., 2019, Moe et al., 2018). Our findings in relation to injury severity are therefore in line with the existing literature, and may reflect lesions as such, but possibly also atrophy and neurodegenerative processes, considering that this is particularly linked to long-term consequences of white matter pathology after TBI (Graham & Sharp, 2019). Time since injury ranged from 21 to 575 months in our study. This means that the most pronounced initial injury-related atrophy has typically occurred, but there can still be considerable within-group variability in underlying factors (e.g., neurodegeneration) that may be associated with further progressive brain volume loss (Graham & Sharp, 2019).

In contrast to the robust findings related to change in self-reported everyday cognitive control function during treatment, there were no statistically significant associations between regional brain volume and the baseline BRIEF-A GEC score. The only statistically significant effect for the baseline measures of functioning was found for the cognitive control efficacy composite score, with the largest clusters revealing a commonly observed association between larger regional brain volume in both gray- and white matter and more efficient (i.e., faster) cognitive control processing. Performance-based and self-report measures of cognitive control function capture overlapping, but distinct phenomena (Løvstad et al., 2012). Moreover, performance-based measures of cognitive control function are thought to reflect *optimal performance* (Toplak et al., 2013)*,* and are therefore more likely to be directly linked to the brain's structural integrity compared to self-reported cognitive control measures, which are thought to reflect *typical performance* (Toplak et al., 2013)*,* i.e., how the persons experience their function in daily life, which again is affected by personal and contextual mediating factors. Current findings in cognitive rehabilitation after brain injury support superior efficiency of targeting *typical functioning* through psychoeducation and compensatory strategies*,* rather than restitutional training of specific skills (i.e.*, optimal performance, ‘training the brain as a muscle’*) (Tate et al., 2014). In line with prior studies, we found limited change in the performance-based cognitive control efficacy measure during treatment (Tornås et al., 2016b). There were also no statistically significant associations between regional brain volume and change in cognitive control efficacy during treatment. Our study therefore indicates that the response to cognitive rehabilitation targeting *typical function* is accompanied by unique patterns of regional brain volume at baseline. However, the lack of multiple baseline measurements to control for practice effects in the performance-based measures limits the validity of this interpretation, and future studies should aim to further disentangle this potential dissociation.

A strength of our approach is that the analyses account for visible lesions. However, lesion mapping was based on T1 scans which are not particularly sensitive to TBI pathology in general and TAI in particular (Skandsen et al., 2020). Dealing with lesions in advanced MRI analyses is a huge challenge in TBI research in general (Olsen et al., 2021), and no single neuroimaging method is sufficient for full characterization and phenotyping of TBI (Amyot et al., 2015). Future studies may benefit from integrating information from a wider range of clinical MRI sequences such as fluid attenuated inversion recovery (FLAIR) and susceptibility weighted imaging (SWI) in order to more precisely capture acute and subacute pathology (Sørensen & Moen, 2020). Moreover, our study focused on TBM based measures of brain structure. Other studies have for example shown that diffusion tensor imaging (DTI) is particularly sensitive in detecting associations between white matter organization and performance-based cognitive control dysfunction (Håberg et al., 2015), but that BOLD fMRI is more sensitive in capturing compensatory functional adaptations in the brain that are linked to self-reported everyday cognitive control function after moderate/severe TBI (Olsen et al., 2015). Interestingly, preliminary results from a small sample with different types of ABI indicate that baseline functional brain network modularity is associated with improvement in attention and executive function after cognitive training (Arnemann et al., 2015). Despite the increased complexity in data analysis, future imaging studies in cognitive rehabilitation after TBI may therefore benefit from taking a multimodal approach including measures of both brain structure and function.

Our study provides important proof of concept that regional brain volume at study baseline is linked to treatment outcome after cognitive rehabilitation in TBI. The study was based on data from an RCT and applied a robust selection of outcome measures. The original trial included patients with different ABI etiologies, but the current study focused on a subsample of patients with TBI. This was done to obtain increased control of etiological factors and pathological processes affecting the neuroimaging findings, which strengthens the internal validity of the findings, but limits the generalizability to populations with other types of ABI. Collapsing interventions that differed in content and theoretical foundation was also necessary to increase statistical power. This could be justified as both treatment groups had a significant improvement in the main outcome measure during treatment (Tornås et al., 2016a). Our results therefore reflect structural brain measures that are linked to a *general* response to cognitive rehabilitation at a *group level*. Although comparing favorably to most existing neuroimaging studies in cognitive rehabilitation after brain injury (Caeyenberghs et al., 2018), the sample size was modest, and was, like other TBI studies, characterized by considerable heterogeneity in injury severity and time since injury. Importantly, the main results were quite robust when accounting for some of this variance statistically, but future larger studies will have the benefit of more closely mapping such effects. Future studies should aim to investigate the imaging-based predictive value of responding to *specific* treatments at an *individual level*. Considering the heterogeneity in pathology and outcomes in TBI, and the considerable researcher degrees of freedom in MRI data analysis (Nichols et al., 2017), future imaging studies in cognitive rehabilitation of TBI should be pre-registered for transparency.

The important clinically relevant question of *what works for whom*, and *why*, in the context of cognitive rehabilitation after TBI is still largely unanswered. Here, we provide preliminary evidence that TBM-based regional brain volume at baseline is associated with treatment response. Particularly strong candidate structural brain measures with relevance for rehabilitation of cognitive control function after TBI were found in midline fronto-parietal regions, including the anterior and posterior cingulate cortices. Future pre-registered larger-scale trials should determine the added value of multimodal imaging parameters for predicting treatment response and patient stratification in cognitive rehabilitation after TBI.

## CRediT authorship contribution statement

**Alexander Olsen:** Conceptualization, Methodology, Formal analysis, Investigation, Data curation, Writing – original draft, Writing – review & editing, Visualization. **Emily L. Dennis:** Conceptualization, Methodology, Formal analysis, Investigation, Writing – original draft, Writing – review & editing, Visualization. **Jan Stubberud:** Conceptualization, Methodology, Investigation, Writing – original draft, Writing – review & editing. **Elizabeth S. Hovenden:** Formal analysis, Investigation, Data curation, Writing – review & editing, Visualization. **Anne-Kristin Solbakk:** Conceptualization, Methodology, Writing – review & editing. **Tor Endestad:** Conceptualization, Writing – review & editing. **Per Kristian Hol:** Formal analysis, Investigation, Data curation, Writing – review & editing. **Anne-Kristine Schanke:** Conceptualization, Methodology, Writing – review & editing. **Marianne Løvstad:** Conceptualization, Investigation, Writing – original draft, Writing – review & editing. **Sveinung Tornås:** Conceptualization, Methodology, Formal analysis, Investigation, Data curation, Writing – original draft, Writing – review & editing.

## Declaration of Competing Interest

The authors declare that they have no known competing financial interests or personal relationships that could have appeared to influence the work reported in this paper.
